# The Utility of Extracorporeal Membrane Oxygenation in Patients With Hematologic Malignancies: A Literature Review

**DOI:** 10.7759/cureus.9118

**Published:** 2020-07-10

**Authors:** Praveena Tathineni, Mauna Pandya, Bassem Chaar

**Affiliations:** 1 Internal Medicine, University of Illinois at Chicago/Advocate Christ Medical Center, Chicago, USA; 2 Hematology and Oncology, Advocate Christ Medical Center, Chicago, USA; 3 Hematology and Oncology, University of Illinois at Chicago/Advocate Christ Medical Center, Chicago, USA

**Keywords:** ecmo, malignant hematology, oncology, chemotherapy, lymphoma

## Abstract

Extracorporeal membrane oxygenation (ECMO) is used to provide respiratory and/or circulatory support for critically ill patients. In people suffering from hematologic malignancies (HMs), acute respiratory failure often necessitates intensive care. Whereas initial studies reported that these patients generally have poor outcomes, studies conducted within the last 10 years have shown that ECMO is quite beneficial for patients with HMs. This review showcases data from 2010 to 2019 demonstrating the utility of ECMO in cancer patients. Retrospective studies revealed long-term disease-free survival, particularly when ECMO served as a bridge through chemotherapy. Case reports suggested strong evidence of mortality benefit from ECMO, especially in patients with aggressive lymphomas. However, a systematic approach is needed to better quantify and validate these findings. Studies with larger sample size and prospective cohorts are needed to help create well-defined guidelines for physicians approaching the treatment of cancer patients on ECMO.

## Introduction and background

Extracorporeal membrane oxygenation (ECMO) is a device used to drain blood from the venous system, oxygenate it with an artificial filter, and return it to the venous or arterial supply - acting as a cardiopulmonary bypass [1]. During the last decade, the use of ECMO in the intensive care unit (ICU) has exponentially increased. It is generally indicated for patients suffering from acute respiratory distress syndrome (ARDS), and/or cardiogenic shock that is refractory to maximal medical management, including inotropic support and/or placement of an intra-aortic balloon pump [2]. Historically, ECMO has been used to provide both circulatory and respiratory support. Veno-arterial (VA) ECMO is used in cardiogenic shock to maintain a patient’s systemic circulation, functioning as a bridge to myocardial tissue recovery, destination therapy with left ventricular assist device placement or orthotopic heart transplant, or coronary artery bypass surgery [2]. It involves inserting a percutaneous cannula to drain blood from the inferior vena cava to the ECMO circuit and deliver newly oxygenated blood to the aorta via the femoral artery [3]. Veno-venous (VV) ECMO involves inserting a large bore cannula in the femoral vein or internal jugular vein to drain blood from the inferior vena cava and pass it through the ECMO circuit for oxygenation and removal of carbon dioxide. The filtered blood is returned to the right atrium via the superior vena cava, thus helping maintain gas exchange [3].

In patients with hematologic malignancies (HMs) such as leukemia, lymphoma, and multiple myeloma, acute respiratory failure is the most common diagnosis warranting ICU admission [4]. While recent advances in chemotherapy and hematopoietic stem cell transplant (HSCT) have benefited these patients, up to 50% of those hospitalized eventually require mechanical ventilation [5-6]. Under such circumstances, ECMO may be used for severe circulatory and/or pulmonary dysfunction [7]; it is also used in patients with tumor lysis syndrome to help stabilize them through chemotherapy [8]. The complex nature of patients with HMs increases their risk of complications from ECMO, specifically bleeding and infections [9-11]. The additional use of chemotherapy often leads to cytopenias and extended periods of marrow aplasia, making them susceptible to bacterial, viral, and fungal infections. Hence, initiating ECMO in patients with HMs has been associated with a high-risk state [9-11].

Studies conducted in the late 20th century imply that patients with HMs on ECMO have poor outcomes [12-18]. More recent analyses have demonstrated long-term disease-free survival in patients with HMs on ECMO [4, 9]. This association of ECMO with mortality benefit in patients with HMs poses the following question: how should hematologists/oncologists manage aggressive malignancies while patients are on ECMO? A comprehensive review comprising findings from the last decade on this topic has not yet been published. The purpose of this article is to gather and analyze data from 2010 to 2019 regarding the use of ECMO in patients with HMs, and its relation to mortality. We believe that the complexity of disease in this subset of patients necessitates clearer and more defined treatment protocols for patients with HMs who require ECMO.

## Review

Search strategy

The search was conducted using the PubMed database and included case reports, case series, reviews, and studies. The following keywords were used: extracorporeal membrane oxygenation, malignant hematology, oncology, chemotherapy, and lymphoma. We screened available articles from 2010 to 2019 that discussed the use of ECMO in patients suffering from aggressive HMs. This was followed by a search using phrases such as “successful treatment with chemotherapy delivered under ECMO,” often accompanied by the keyword ‘lymphoma.’ Our search was completed by analyzing pharmacological dosing for patients on ECMO who were treated with chemotherapy.

Reports of patients with HMs who required ECMO for pulmonary support

At the start of the decade, studies involving a variety of adult cancer patients were conducted to assess the effect of extracorporeal life support on clinical outcomes. One study from 2010 included 47 solid tumor patients, 21 patients with HMs, and four post-HSCT patients - all of whom required ECMO for pulmonary support. Ultimately, 61% of this group died on ECMO, 32% survived to hospital discharge, and 7% survived on ECMO but died before discharge [16]. This supported the general sentiment at the time that the use of ECMO in cancer patients is rarely successful from a mortality perspective. Kang et al. performed a systematic retrospective review of clinical outcomes in 15 patients with HMs who were treated with ECMO after failure of optimal conventional therapy [17]. This study was conducted in South Korea; two-thirds of the patients had a primary diagnosis of acute myelogenous leukemia or acute lymphoblastic leukemia. Their outcomes were compared to 33 immunocompetent patients with documented cardiorespiratory failure that warranted ECMO support. The patients with underlying HMs were associated with significantly higher mortality rates compared to the immunocompetent patients, partially attributed to infections and hyperbilirubinemia during ECMO [17].

In 2012, Azoulay et al. performed a prospective, observational cohort study in 17 centers in France and Belgium to assess outcomes in critically ill malignant hematology patients [9]. Amongst 1,011 patients with HMs admitted to the ICU, the mortality rate was 39.3%. The subsequent disease-control rate six months after discharge from the hospital was 80% [9]. Although this study did not specifically take ECMO into account, the mortality rate was significantly lower than that established in prior studies. This was a starting point for the conclusion and realization that critically ill patients with HMs may have improved survival rates [9].

In 2014, Wohlfarth et al. conducted a retrospective cohort study of 14 adult Austrian patients with HMs, all of whom received ECMO support due to acute respiratory failure [4]. They had a variety of aggressive HMs, including non-Hodgkin lymphoma, Hodgkin lymphoma, acute myeloid leukemia, and multiple myeloma. Three patients received VA ECMO and the remaining 11 patients received VV ECMO. After being on ECMO for an average of 8.5 days, 50% of the patients survived the ICU and hospital stay. Their long-term survival was 100% after a median follow-up of 36 months post discharge [4]. This report was the first of its kind and implied that patients with HMs undergoing ECMO for severe acute respiratory failure may have favorable long-term outcomes.

This accumulated research shows differing conclusions for patients with HMs requiring ECMO for pulmonary support. Whereas studies published earlier suggested that these patients have a mortality rate greater than 50%, studies from 2014 onward suggest they have a mortality rate less than 50%. The progression in the literature shows a general transition from seeing poor outcomes for patients with HMs on ECMO to strong outcomes with long-term survival. Although an extensive search was conducted to find more data specifically on patients with HMs on ECMO, it resulted in a limited number of articles. Additional studies are required to draw more reliable conclusions. In addition, three of the four studies mentioned above were retrospective analyses, limiting the ability to control for several potential variables. This calls for systematic prospective studies in the future with larger sample size.

Reports of patients with HMs who were successfully placed on ECMO as a bridge to chemotherapy

Due to the adverse effects of ECMO, specifically infections, bleeding, and thrombocytopenia, the administration of chemotherapy is not routinely advised in those patients [18]. According to the Extracorporeal Life Support Organization (ELSO), an international nonprofit organization who maintains the worldwide registry of ECMO in active ELSO centers, there are no absolute contraindications to administering chemotherapy while on ECMO [19]. However, ELSO does suggest using extra precautionary measures in patients greater than 65 years of age, and/or those with an absolute neutrophil count of less than 400/mm^3^ [19-20]. The following cases serve as examples of successful treatment with chemotherapy in patients with HMs requiring ECMO, specifically those with high-grade lymphomas.

First, Aboud et al. described the case of a 43-year-old woman with newly diagnosed aggressive non-Hodgkin’s lymphoma who was successfully placed on ECMO as a bridge to curative therapy [21]. Due to massive tumor burden compromising the trachea, she required VV ECMO for imminent hypoxic respiratory failure. While on ECMO, she was treated with lymphoma-specific chemotherapy (Cyclophosphamide, Doxorubicin, Vincristine, Prednisolone, and Rituximab) [21]. The patient was successfully weaned from ECMO six days after admission and was discharged home after a 36-day stay in the ICU. Her subsequent pulmonary function tests were normal, and she achieved complete remission [21].

Next, Worku et al. reported the case of a 41-year-old woman who initially presented with dyspnea due to a 14-cm anterior mediastinal mass encasing the superior vena cava and heart [22]. She was diagnosed with high-grade diffuse large B-cell lymphoma and was started on lymphoma-specific chemotherapy (Dexamethasone, Etoposide, Adriamycin, and Vincristine). Shortly after, she developed cardiogenic shock requiring emergent intubation and intra-aortic balloon pump placement [22]. Her disease-specific prognosis was favorable, considering that diffuse large B-cell lymphoma is typically responsive to appropriate systemic therapy [22]. Hence she was initiated on VA ECMO as a bridge to treatment, and received a five-day course of chemotherapy while on extracorporeal life support. ECMO was discontinued after one cycle of chemotherapy, she was decannulated, and she eventually regained complete autonomous function of her cardiopulmonary circuit. She completed six cycles of chemotherapy and was discharged to a rehabilitation facility after a 144-day hospital stay [22].

Furthermore, Oto et al. described the case of a 40-year-old male with chest pain and dyspnea due to a 12 cm by 7 cm mediastinal mass [23]. He underwent tracheal intubation followed immediately by urgent ECMO placement as the tumor completely obstructed the left main bronchus. Fine needle biopsy of the mediastinal tumor was performed, diagnosing precursor T lymphoblastic lymphoma [23]. Chemotherapy with Adriamycin, Vincristine, Cyclophosphamide, and Prednisolone was initiated on hospital day 3 for tumor debulking, after which the mediastinal mass rapidly decreased in size. This was associated with an improvement in oxygenation, and he was weaned from ECMO on hospital day 8 [23]. Subsequent consolidation therapy was administered during his hospital stay, and partial remission of the tumor was confirmed on hospital day 87. The patient proceeded to HSCT with favorable prognosis [23].

Lastly, Allain et al. described the case of a 65-year-old male who was transferred to the ICU for cardiogenic shock, and soon thereafter was placed on ECMO [24]. A diagnosis of high-grade B cell lymphoma was made and he was started on emergent chemotherapy with Cyclophosphamide, Vindesine, and Solumedrol on day 8 of ECMO support. He was weaned from mechanical support on day 11, and discharged home after a 60-day hospital stay. Six months after discharge, complete remission was confirmed [24]. Without ECMO support, this patient would likely not have recovered from his aggressive B cell lymphoma.

The various examples described above demonstrate that ECMO can serve as a bridge to life-saving, and even curative chemotherapeutic options [21-24]. ECMO can help provide patients the hemodynamic stability they need in order to receive systemic therapy, and thus achieve a long-term disease-free state. In patients with treatment-responsive HMs who are hemodynamically stable on ECMO, and who can tolerate chemotherapy, we suggest that chemotherapy should be considered and administered. The benefits of undergoing ECMO often do outweigh the risks in these patients and can result in mortality benefit, a decision that needs to be assessed on a case-by-case basis. Each patient must be evaluated individually, and comprehensively. Although most of the case reports mentioned above reported positive outcomes, they are still anecdotal studies and are likely subject to bias. There is a need for systematic trials, including prospective studies, which can help provide statistical significance and power to the conclusions made in the case reports we reviewed.

Pharmacokinetics of chemotherapy delivered under ECMO

The pharmacokinetics of chemotherapy delivered under ECMO has not been sufficiently studied based on the literature review done on this topic. A review article written by Sherwin et al. did establish pharmacokinetic data for anti-infective drugs administered to patients on ECMO [25]. Pharmacokinetics are known to be affected by ECMO in three main ways: 1) direct extraction by the circuit itself, 2) changes in volume of distribution (often increased due to drug extraction and hemodilution in the patient), and 3) altered clearance of the drug (related to renal dysfunction) [25]. Through retrospective data analysis, it was noted that the volume of distribution and clearance of drugs is unchanged in the adult population. Pharmacotherapy dosing recommendations for adults on ECMO are similar to those not requiring ECMO [25]. However, most data on this subject is derived from neonatal studies and case series on adult patients without critically ill controls. The authors have called for further systematic investigation in adult patients to determine better, the proper dosing recommendations in this patient population [25].

Additional data is needed in order to make conclusive recommendations on chemotherapy dosing in patients with HMs who require extracorporeal life support. As ECMO technology is rapidly evolving and being utilized more often in critically ill patients across the world, randomized controlled trials regarding pharmacokinetics of chemotherapy on mechanical support are warranted and desperately needed. These studies are necessary and would contribute to improved treatment of critically ill oncology patients, optimizing outcomes while minimizing toxicity and treatment-related mortality.

## Conclusions

ECMO is often used in critically ill patients with acute respiratory failure. Differing conclusions regarding its use in patients with concurrent HMs have been reported. Studies published in the late 20th and early 21st century concluded that patients with HMs on ECMO have poor outcomes. More recent studies published during 2010 to 2019 have suggested a mortality benefit in this patient population, as a result of ECMO support. Reports from 2014 to 2019 have particularly implied that patients with HMs requiring ECMO have an increasingly favorable prognosis, some even exhibiting long-term survival. However, this data consists of small retrospective studies and case reports. Large-scale retrospective and prospective clinical studies should be conducted to establish a stronger conclusion regarding the mortality benefit of ECMO for patients with HMs. Randomized controlled trials studying the pharmacokinetics of chemotherapy in patients on ECMO are also needed to help clinicians reliably dose antineoplastic agents, optimizing outcome and decreasing toxicities. As ECMO becomes more available and continues to gain support in the care of the critically ill, we believe it is important for guidelines to be created to help in the treatment decisions of those patients.
